# The effects of oxygen addition on microstructure and mechanical properties of Ti-Mo alloys for biomedical application

**DOI:** 10.3389/fbioe.2024.1380503

**Published:** 2024-03-28

**Authors:** Sengo Kobayashi, Satoshi Okano

**Affiliations:** Graduate School of Science and Engineering, Ehime University, Matsuyama, Japan

**Keywords:** titanium alloy, oxygen, microstructure, mechanical property, biomedical application, strength, phase transformation

## Abstract

The effective use of oxygen as an alloying element in Ti alloys is attractive due to the reduction of production cost and the increase in strength and hardness of the alloy. Although the oxygen addition in a Ti alloy increases strength and hardness, it may induce brittleness. An appropriate combination of alloying elements and thermomechanical treatment must be clarified for the use of oxygen as an alloying element. Ti-(0, 1.0, 2.0, 3.0)Mo-(0, 1.5, 3.0)O alloys were developed, and their microstructure and mechanical properties were examined. Ti-1Mo-3O alloy exhibited fine grains of α+β two phases having the tensile strength of 1,297 MPa with 15.5% for total strain at fracture. The Ti-1Mo-3O alloy has 1.5 times the tensile strength and the same total strain as the Ti-6Al-4V ELI alloy. Ti-(1.0, 2.0, 3.0)Mo-1.5O alloys also have excellent mechanical properties, with tensile strength of about 1,050–1,150 MPa and a total strain of about 20%–25%. In order to develop a high strength and moderate ductility Ti-Mo alloy using oxygen as an alloying element, the microstructure should have fine grains of α+β two phases with proper volume fraction of α and β phases and specific molybdenum concentration in β phase.

## 1 Introduction

Titanium alloys, such as Ti-6Al-4V ELI, Ti-13Nb-13Zr, and Ti-15Mo (in mass%), have been used as biomedical materials due to their proper mechanical properties, excellent corrosion resistance, and high biocompatibility with non-magnetic (Kaur and Singh, 2019; Sarraf et al., 2022; Marin and Lanzutti, 2023; Pesode and Barve, 2023). Although titanium alloys are widely used in biomedical fields, they are expensive for refining, processing, and improving mechanical properties. Many expensive rare elements such as V, Nb, and Mo are used to improve the mechanical properties of Ti alloys. Therefore, the development of a Ti alloy using inexpensive, ubiquitous elements, such as O, N, C, and Fe, has been actively carried out (Niinomi, 2011; Song et al., 2023). For example, the Ti-Fe-O-N alloys, which were strengthened by the solid solution strengthening of oxygen and nitrogen, were developed (Fujii et al., 2002; Fujii et al., 2003). In addition, various peculiar phenomena were examined in oxygen-added Ti-Nb-based shape memory and superelasticity alloys (Tahara et al., 2011). Adding oxygen can significantly increase strength and hardness while reducing ductility (Yu et al., 2015). Oxygen addition also changes the microstructure in Ti alloys. The oxygen addition suppressed the martensitic transformation of β→α' and α" phase (Kawano et al., 2019). It is interesting to note that oxygen addition suppressed the β→athermal ω phase transformation during quenching while it accelerated the β→isothermal ω phase transformation during slow cooling (Kobayashi et al., 2020a) or isothermal holding (Niinomi et al., 2016). Examined the effects of oxygen or nitrogen addition on the age-hardening and spinodal decomposition of α′ or α" phase in Ti-4Mo (Saeki et al., 2018) and Ti-10V-(0, 1, 3)O or N alloys (Kobayashi et al., 2020b). The increment of age-hardening was decreased as oxygen or nitrogen content in the alloy increased. The spinodal decomposition wavelength (λp) in Ti-10V alloy was increased by adding oxygen or nitrogen. The increment of the λp by the nitrogen addition was larger than that in the case of oxygen addition due to the elastic energy of spinodal decomposition in nitrogen-containing alloys being much larger than that in oxygen-containing alloys. The effects of oxygen addition on the microstructure formation and mechanical properties have been gradually clarified. However, the microstructure control to achieve high strength with good ductility of Ti alloy with oxygen has not been well understood. Therefore, in the present study, we examined the effects of oxygen addition on the microstructure and mechanical properties of hot-rolled Ti-Mo alloys to determine the strategy for developing high-strength and good ductility oxygen-containing Ti alloys.

## 2 Experimental procedures

Ti-(0, 1.0, 2.0, 3.0) at%Mo-(0, 1.5, 3.0)at%O alloy (hereinafter, the composition is written without at% unless otherwise specified) was melted into a 10 g button-shaped ingot by Ar arc melting, using titanium sponge (>99 mass%), molybdenum (Mo) plate (>99.95 mass%), TiO powder (99.9 mass%), and titanium foil (>99.5 mass%). The TiO powder was wrapped in titanium foil and dissolved with the titanium sponge and Mo plate. Before arc melting each sample, the titanium sponge was melted as an oxygen getter, and the sample was inverted eight times to suppress segregation. The oxygen concentration of the melted alloy was measured using an infrared absorption method (LECO: ONH836). The melted button-shaped ingot was hot-rolled at 850 °C up to 75% rolling reduction at a reduction rate of 0.5 mm/pass, and then it was quenched in ice brine. The mirror-polished sample was etched with a mixture of 8% hydrofluoric acid (HF), 20% nitric acid (HNO_3_), and 72% pure water (H_2_O) in volume ratio, and the microstructure was observed using an optical microscope. X-ray diffractometry (XRD) was performed to identify the constituent phase of each sample using the Cu-Kα X-ray source with a tube voltage of 20 kV and a tube current of 15 mA. The fracture surface of each alloy after the tensile test was observed using a scanning electron microscope (SEM) at an accelerating voltage of 20 kV. A thin foil for transmission electron microscopy (TEM) observation was prepared by the twinjet electropolishing using the mixture of perchloric acid, 1-butanol, and methanol in a volume ratio, 1:5:10, at −30°C. TEM was performed at accelerating voltages of 100 kV (JEOL: JEM-100CXII) and 200 kV (JEOL: JEM-2100). The tensile test was conducted at room temperature with an initial strain rate of 8.33 × 10^−4^ (s^-1^). The gauge length and width of the tensile test piece were 12.1 and 2.5 mm, respectively, with a thickness of 1.0 mm. The elongation up to failure was measured using a strain gauge-type extensometer, and the engineering stress and strain were evaluated. Hardness measurements were carried out at room temperature under 1,000 gf load. Average hardness was calculated from five measuring points.

## 3 Results

Table 1 shows the oxygen content of the prepared Ti-(0–3.0)Mo-(0–3.0)O alloys. Pure Ti (Ti) and Ti-Mo binary alloys contained approximately 0.2–0.5 oxygen. Such oxygen contamination was probably attributed to the fact that raw materials included a small amount of oxygen, and oxygen contamination might occur during the alloy melting process. When melting an oxygen-added alloy, we considered such oxygen contamination and adjusted the target composition to obtain the desired nominal composition. We created an alloy with an oxygen concentration close to the desired one with a deviation of approximately 0.2 at%. Therefore, the alloy compositions will be shown in the nominal composition.

**TABLE 1:** Oxygen content of samples used in the present study.

Nominal composition (at%)	Oxygen content (at%)
Ti	0.56
Ti-1.5O	1.61
Ti-3.0O	2.96
Ti-1.0Mo	0.22
Ti-2.0Mo	0.26
Ti-3.0Mo	0.31
Ti-1.0Mo-1.5O	1.62
Ti-2.0Mo-1.5O	1.57
Ti-3.0Mo-1.5O	1.66
Ti-1.0Mo-3.0O	2.87
Ti-2.0Mo-3.0O	2.95
Ti-3.0Mo-3.0O	3.03

### 3.1 Mechanical propery and microstructure of Ti-(0, 1.5, 3.0)O alloys

**FIGURE 1:**
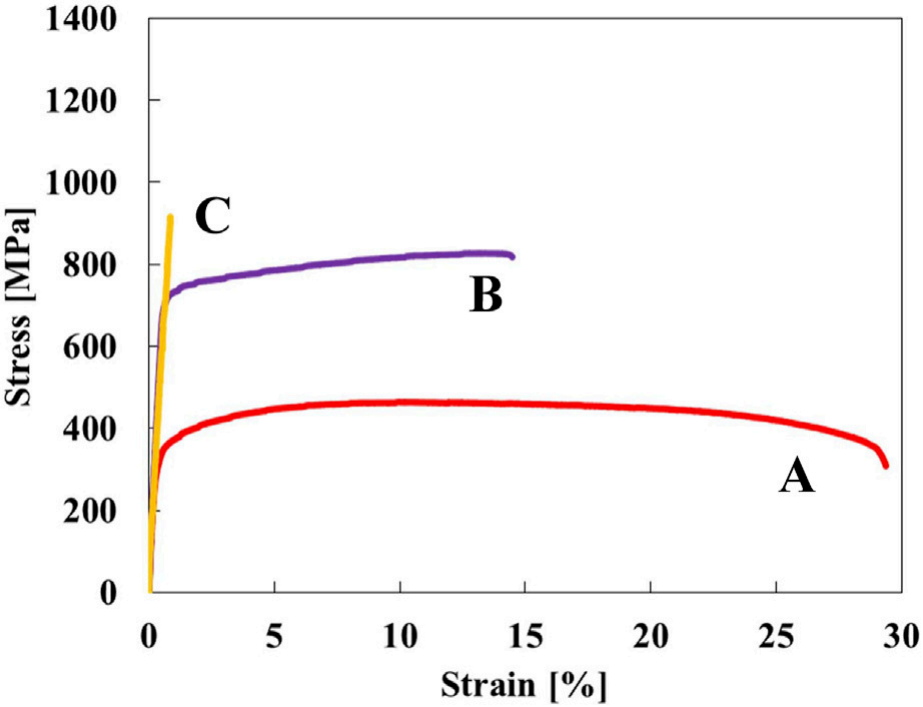
Stress-strain curves for the specimens; (A) Ti, (B) Ti-1.5O, and (C) Ti-3.0O.

**FIGURE 2:**
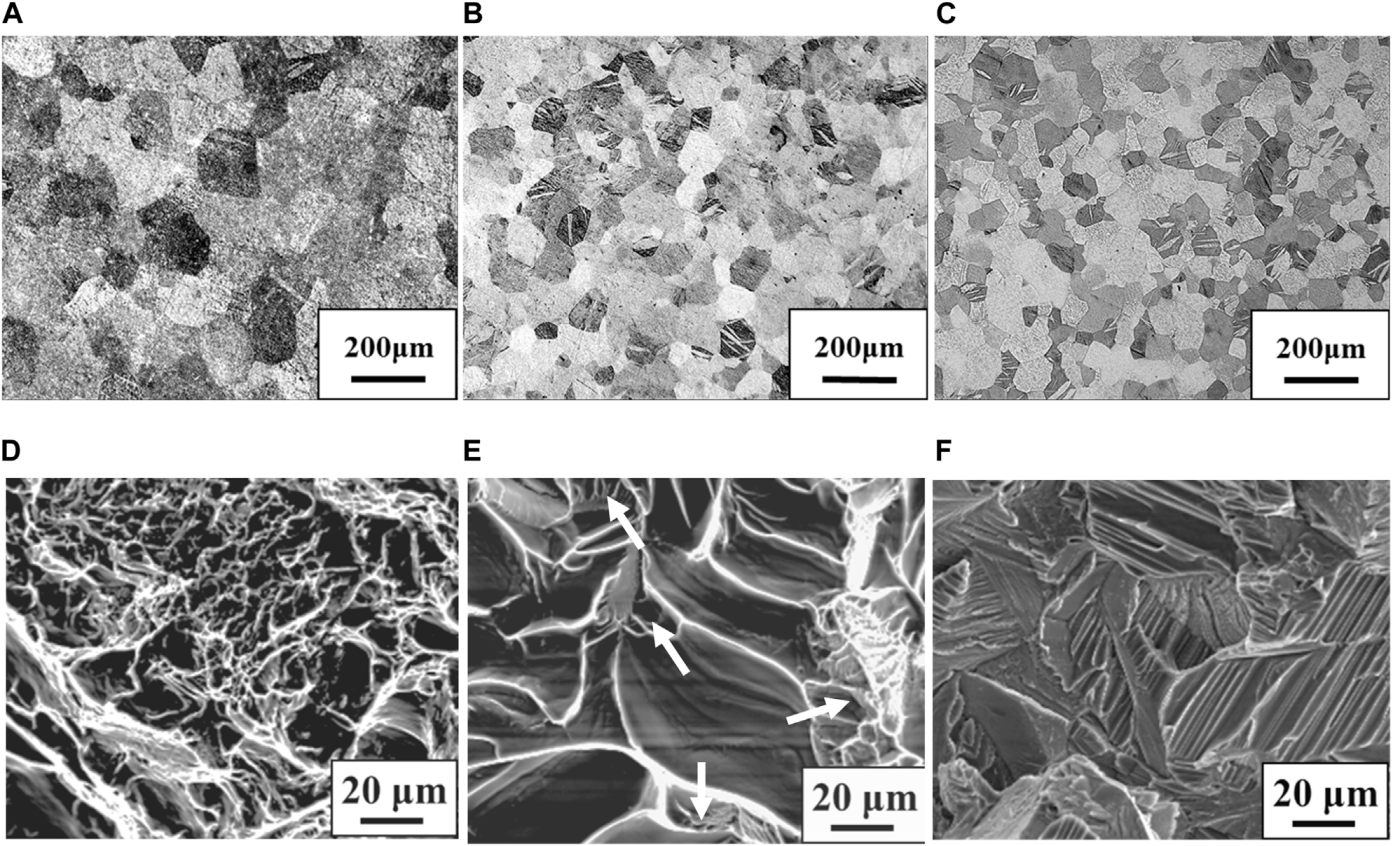
Optical micrographs for the specimens; **(A)** Ti, **(B)** Ti-1.5O and **(C)** Ti-3.0O and scanning electron micrographs for the fracture surface of the specimens; **(D)** Ti, **(E)** Ti-1.5O and **(F)** Ti-3.0O, respectively.

Figure 1 shows stress-strain curves of the Ti-(0, 1.5, 3.0)O alloys. When 1.5 at% oxygen was added to the Ti, the tensile strength increased from 464 MPa to 828 MPa, but the total strain at fracture decreased from 29.2% to 14.5%. The Ti-3.0O alloy fractured during elastic deformation with no plastic elongation. Figures 2A–C are optical micrographs of (A) Ti, (B) Ti-1.5O, and (C) Ti-3.0O alloys, respectively. Equiaxed grains of approximately 50–200 µm in diameter were observed, and the grain size became finer as the amount of oxygen in alloys increased. Figures 2D–F are scanning electron micrographs of the fracture surface of the (D) Ti, (E) Ti-1.5O, and (F) Ti-3.0O alloys, respectively. The dimple pattern of about 5–20 µm in diameter on the fracture surface was observed for the Ti. The dimple size at the fracture surface became large and sharrow, about 20–40 µm in diameter for the Ti-1.5O alloy. But it still had small dimples of about 5–20 µm in diameter, as indicated by arrows. The cleavage surface was observed for the Ti-3.0O alloy, indicating a brittle fracture occurred. The XRD measurements revealed that all Ti-(0, 1.5, 3.0)O alloys were α-phase with hcp structure.

### 3.2 Mechanical propery and microstructure of Ti-(1.0, 2.0, 3.0)Mo alloys

**FIGURE 3:**
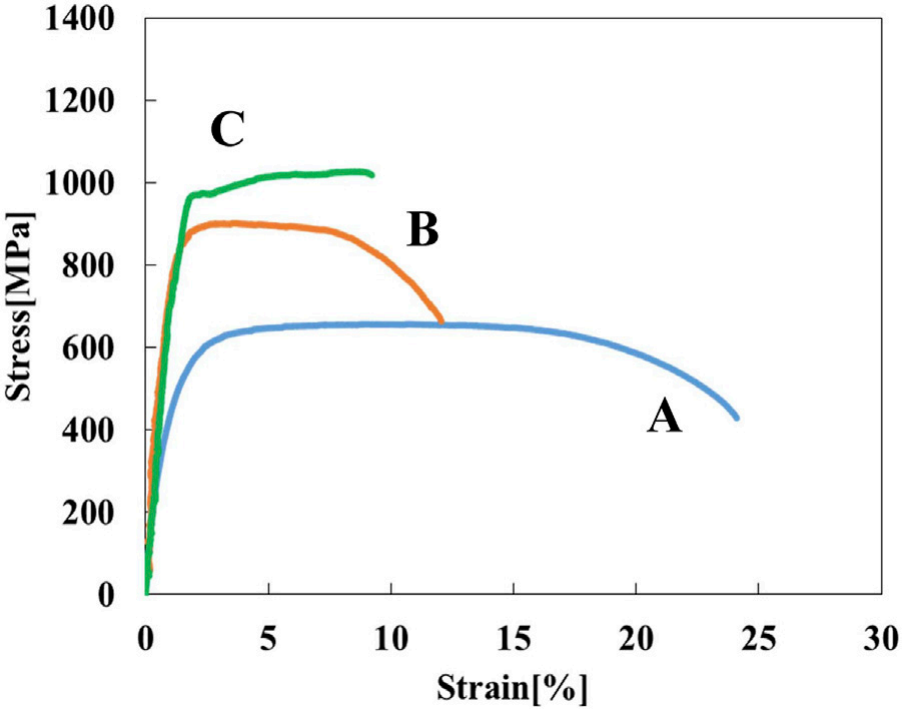
Stress-strain curves for the specimens; (A) Ti-1.0Mo, (B) Ti-2.0Mo, and (C) Ti-3.0Mo.

**FIGURE 4:**
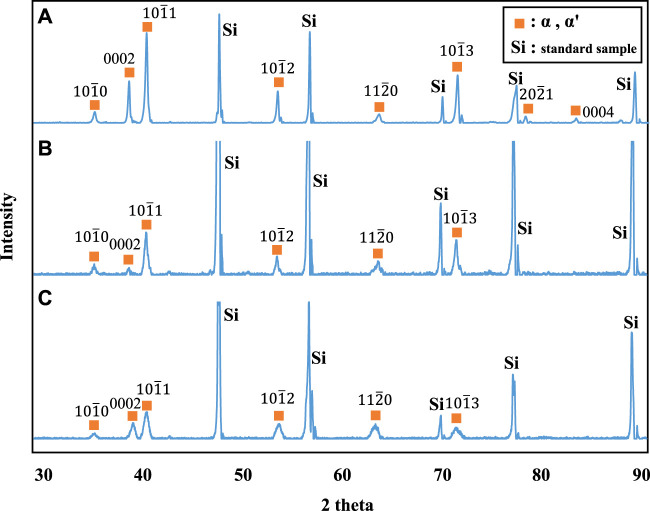
XRD profiles for the specimens; **(A)** Ti-1.0Mo, **(B)** Ti-2.0Mo, and **(C)** Ti-3.0Mo. Simultaneous measurement of silicon powder as a standard sample for the adjustment of the XRD profile also appeared.

**FIGURE 5:**
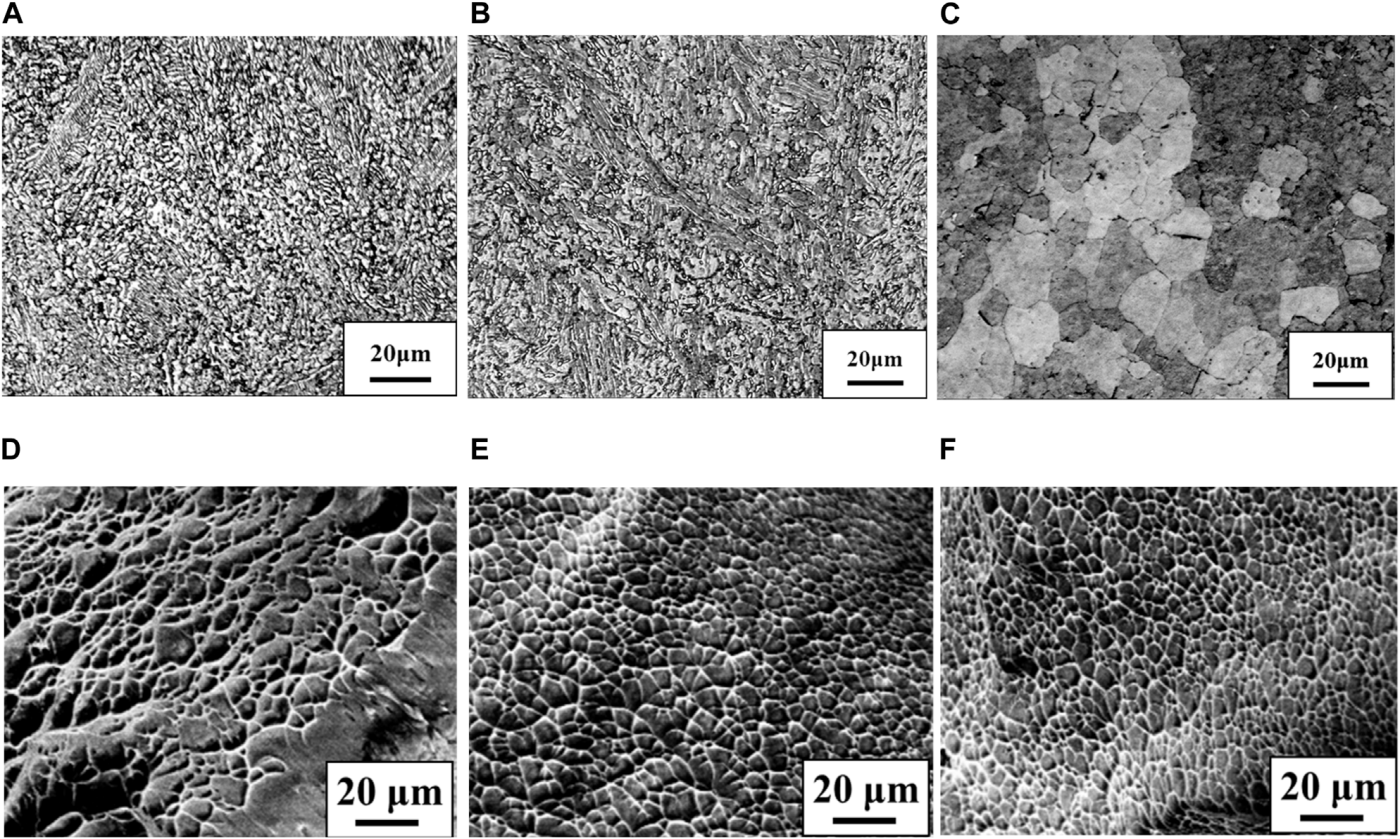
Optical micrographs for the specimens; **(A)** Ti-1.0Mo, **(B)** Ti-2.0Mo and **(C)** Ti-3.0Mo and scanning electron micrographs for the fracture surface of the specimens; **(D)** Ti-1.0Mo, **(E)** Ti-2.0Mo and **(F)** Ti-3.0Mo, respectively.

Figure 3 shows stress-strain curves of the Ti-(1.0, 2.0, 3.0)Mo alloys. The addition of Mo increased the strength and decreased the elongation. The tensile strength increased from 464 MPa of pure Ti to 657, 902, and 1,027 MPa by adding 1.0, 2.0, and 3.0Mo, respectively. On the other hand, the fracture strain decreased from 29.2% of pure Ti to 24.0, 12.0, and 9.1% when the Mo content increased to 1.0, 2.0, and 3.0 at%, respectively. The uniform and local deformations were observed in the Ti-(1.0, 2.0)Mo alloys. XRD measurements shown in Figure 4 revealed that the crystal structure of the Ti-(1.0, 2.0, 3.0)Mo alloys was the α phase or α′ phase with an hcp structure. Figures 5A–C show the optical micrographs of Ti-(1.0, 2.0, 3.0)Mo alloys. Ti-1.0Mo and Ti-2.0Mo alloys were composed of fine grains of several micrometers in size. On the other hand, the Ti-3.0Mo alloy was composed of equiaxed grains with non-uniform sizes of about 5–20 µm in diameter. Figures 5D–F are scanning electron micrographs of the fracture surface of the Ti-(1.0, 2.0, 3.0)Mo alloys. The dimple pattern of about 10 µm in diameter on the fracture surface was observed for all samples. The depth of the dimple, however, seemed to become shallow as the Mo content of the alloys.

### 3.3 Mechanical propery and microstructure of Ti-(1.0, 2.0, 3.0)Mo-1.5O alloys

**FIGURE 6:**
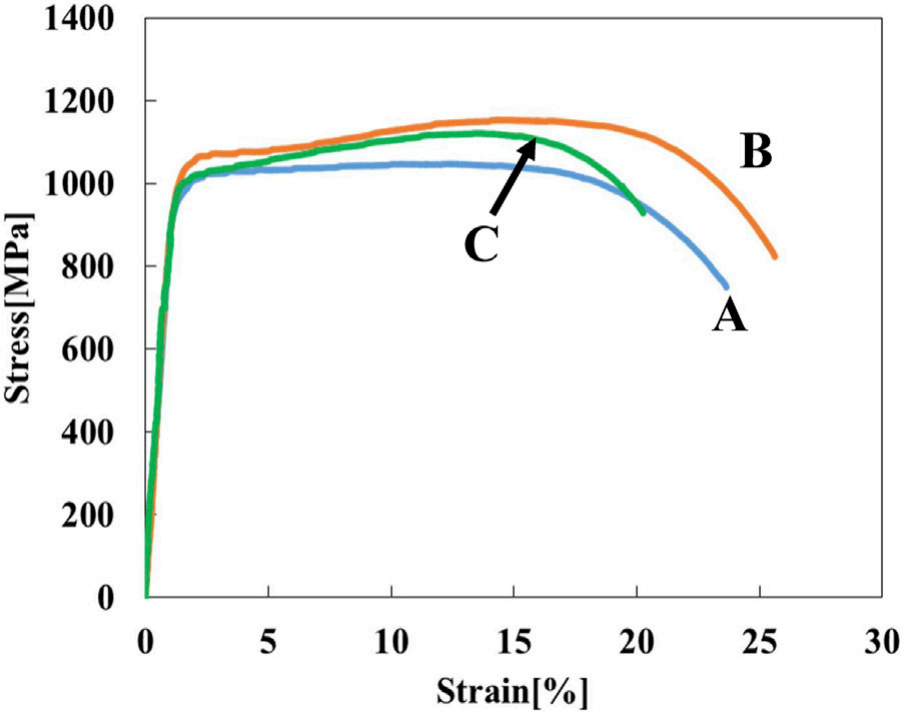
Stress-strain curves for the specimens; (A) Ti-1.0Mo-1.5O, (B) Ti-2.0Mo-1.5O, and (C) Ti-3.0Mo-1.5O.

**FIGURE 7:**
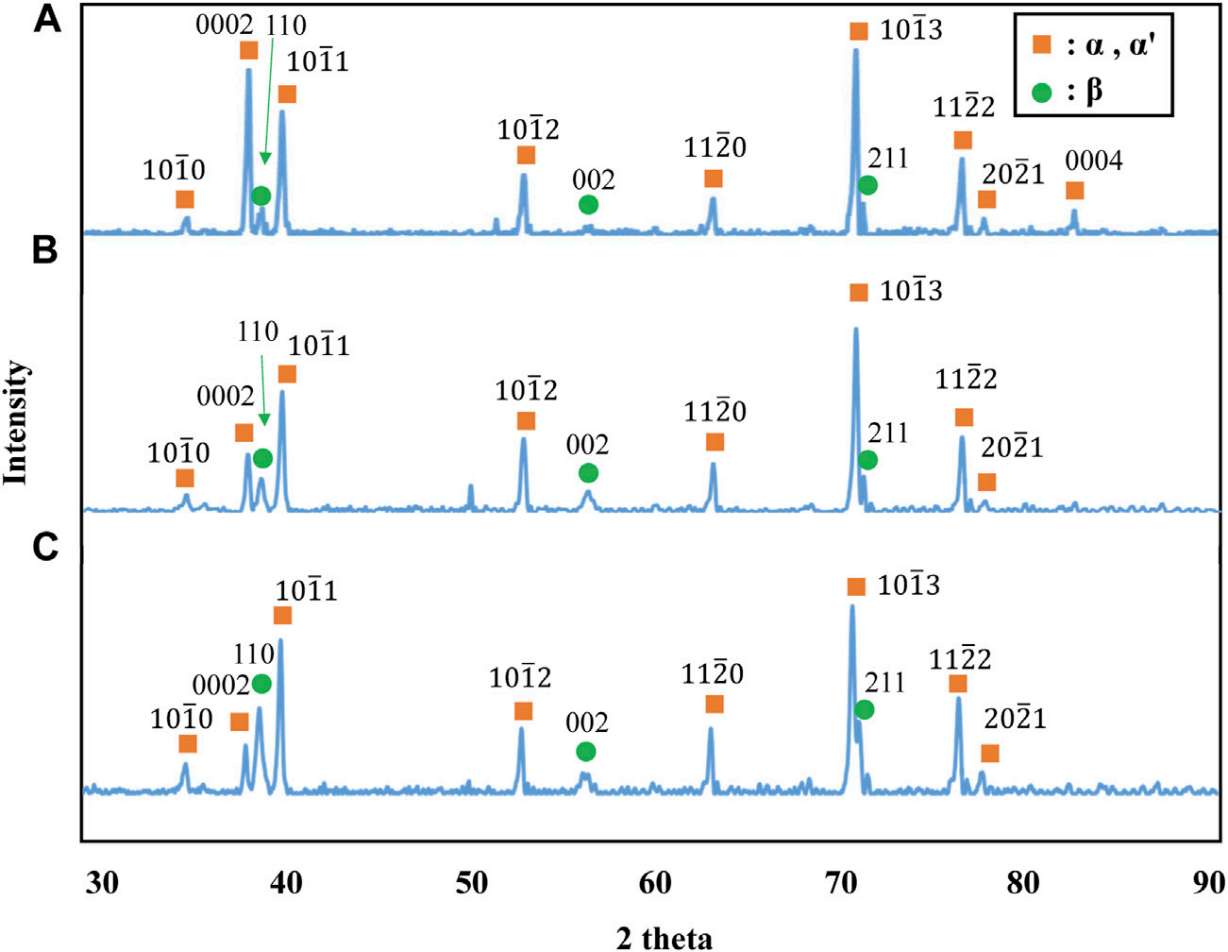
XRD profiles for the specimens; **(A)** Ti-1.0Mo-1.5O, **(B)** Ti-2.0Mo-1.5O, and **(C)** Ti-3.0Mo-1.5O.

**FIGURE 8:**
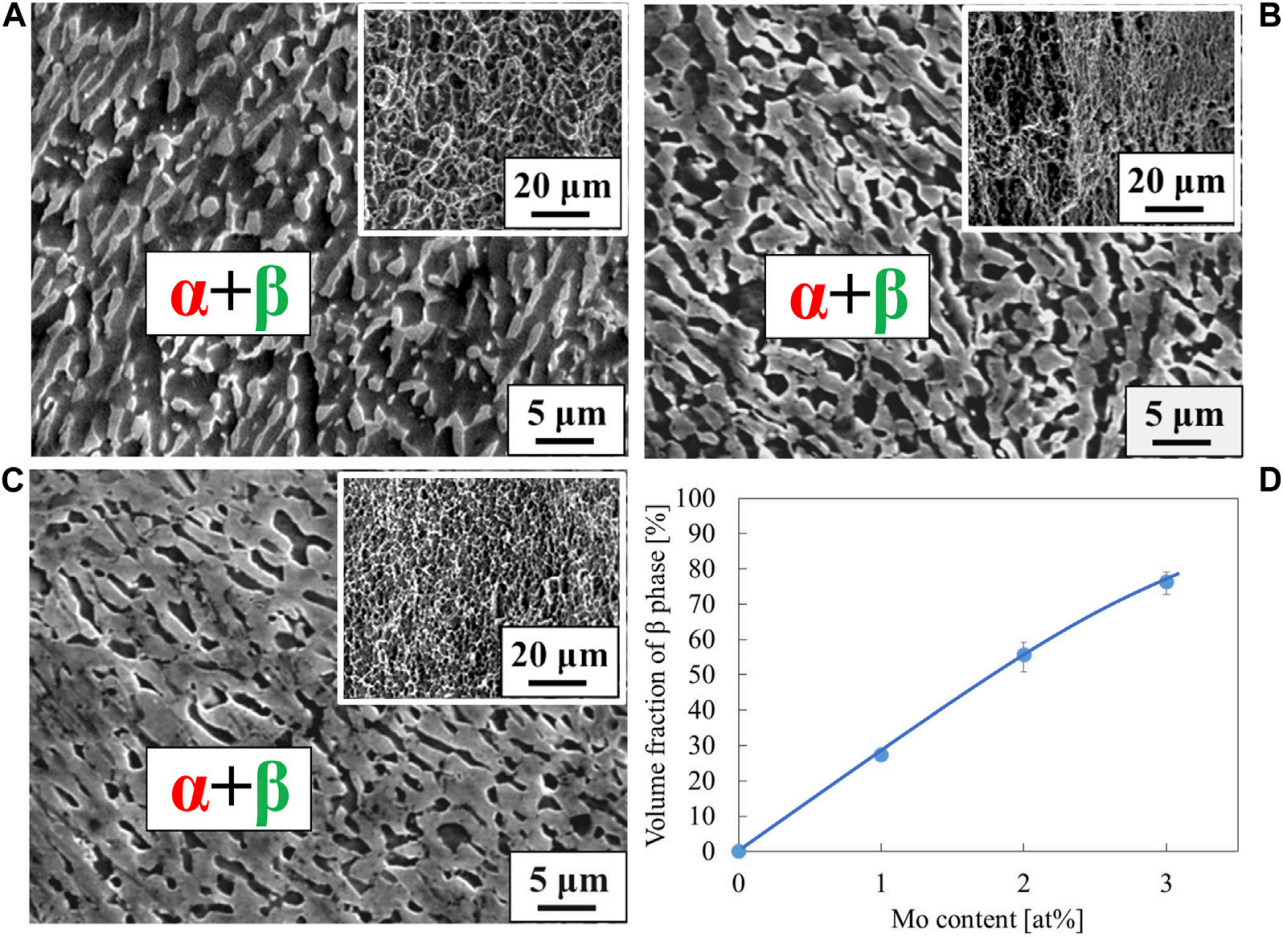
Scanning electron micrographs for the specimens; **(A)** Ti-1.0Mo-1.5O, **(B)** Ti-2.0Mo-1.5O, and **(C)** Ti-3.0Mo-1.5O. The insert image at the upper right corner of each image is a scanning electron micrograph for the fracture surface. The volume fraction of phase was plotted in the inserted graph **(D)**.

The stress-strain curves of Ti-(1.0, 2.0, 3.0)Mo-1.5O alloys are shown in Figure 6. The tensile strength of Ti-1.0Mo-1.5O, Ti-2.0Mo-1.5O, and Ti-3.0Mo-1.5O alloys were 1,048, 1,153, and 1,122 MPa, respectively. By comparing those of Ti-1.0Mo, Ti-2.0Mo, and Ti-3.0Mo alloys, the tensile strength increase for each alloy due to 1.5at% oxygen addition was 391, 251, and 95 MPa, respectively. It should be noted that the total strain of Ti-1.0Mo, Ti-2.0Mo, and Ti-3.0Mo with 1.5 at% oxygen addition were 23.5, 25.6, and 20.2%, showing good uniform elongation accompanied by work hardening. When oxygen or molybdenum was added to the Ti separately, the total strain constantly decreased as the tensile strength increased. However, when 1.5 at% oxygen was added to a Ti-(1.0, 2.0, 3.0)Mo alloys, both tensile strength and total strain increased, except for the total strain of Ti-1.0Mo-1.5O, which was almost the same as that of Ti-1.0Mo alloy. Figure 7 shows the XRD profiles of Ti-yMo-1.5O alloys; (A) y = 1.0, (B) y = 2.0, (C) y = 3.0. Diffraction peaks of α, α′ phase (hcp), and β phase (bcc) were detected in all samples. Figure 8 shows scanning electron micrographs (backscattered electron image: BEI) for the Ti-yMo-1.5O alloys: (A) y = 1.0, (B) y = 2.0, (C) y = 3.0. A fine two-phase structure of several micrometers in diameter was confirmed in all samples. The molybdenum is a β-phase stabilizing element, and it is largely distributed to the β phase in the α+β two-phase mixture. The compositional analysis by the energy dispersive spectroscopy (EDS) revealed that the bright part in the BEI had concentrated Mo and was the β phase. The dark part had a lower Mo concentration than the bright part, which was determined to be the α phase. The graph in Figure 8D shows the variation of the β phase volume fraction in the BEI, indicating that the β phase volume fraction increased as molybdenum content increased. The insert image at the upper right corner of each image is the scanning electron micrograph for the fracture surface of each sample. The fracture surface of each sample exhibited a dimple pattern, whose diameter was almost the same as that of the grain size of α+β two-phase mixture. It should be noted that the dimple size of Ti-(1.0, 2.0, 3.0)Mo-1.5O alloys became much finer than those of Ti-(1.0, 2.0, 3.0)Mo binary alloys, as shown in Figures 5D–F.

### 3.4 Mechanical propery and microstructure of Ti-(1.0, 2.0, 3.0)Mo-3.0O alloys

**FIGURE 9:**
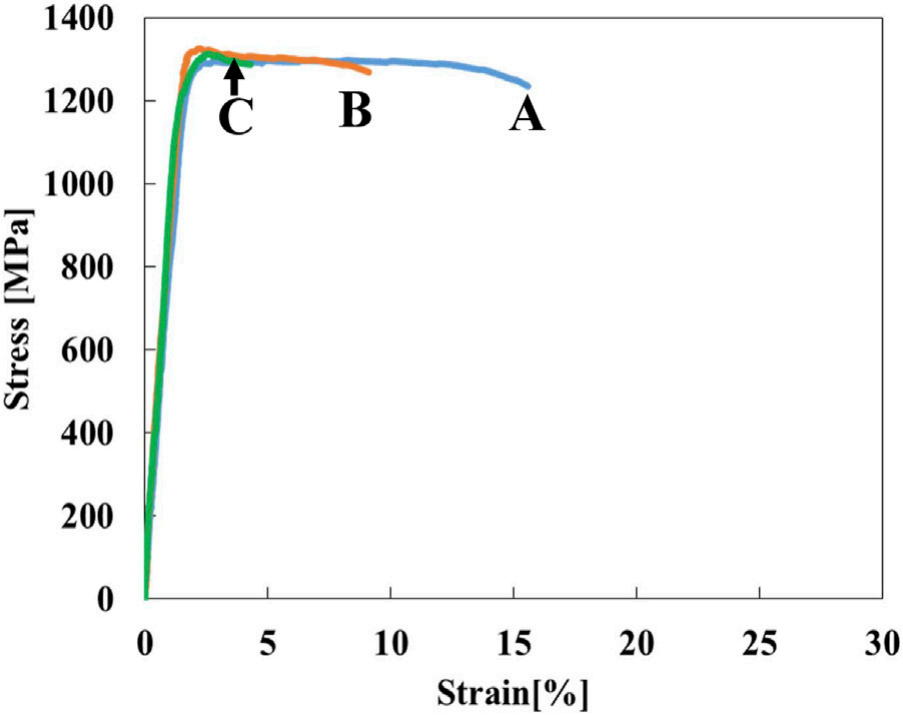
Stress-strain curves for the specimens; (A) Ti-1.0Mo-3.0O, (B) Ti-2.0Mo-3.0O, and (C) Ti-3.0Mo-3.0O.

**FIGURE 10:**
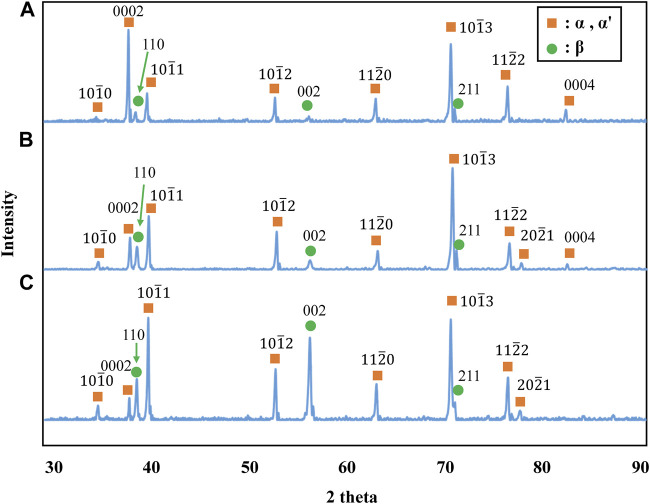
XRD profiles for the specimens; **(A)** Ti-1.0Mo-3.0O, **(B)** Ti-2.0Mo-3.0O, and **(C)** Ti-3.0Mo-3.0O.

**FIGURE 11:**
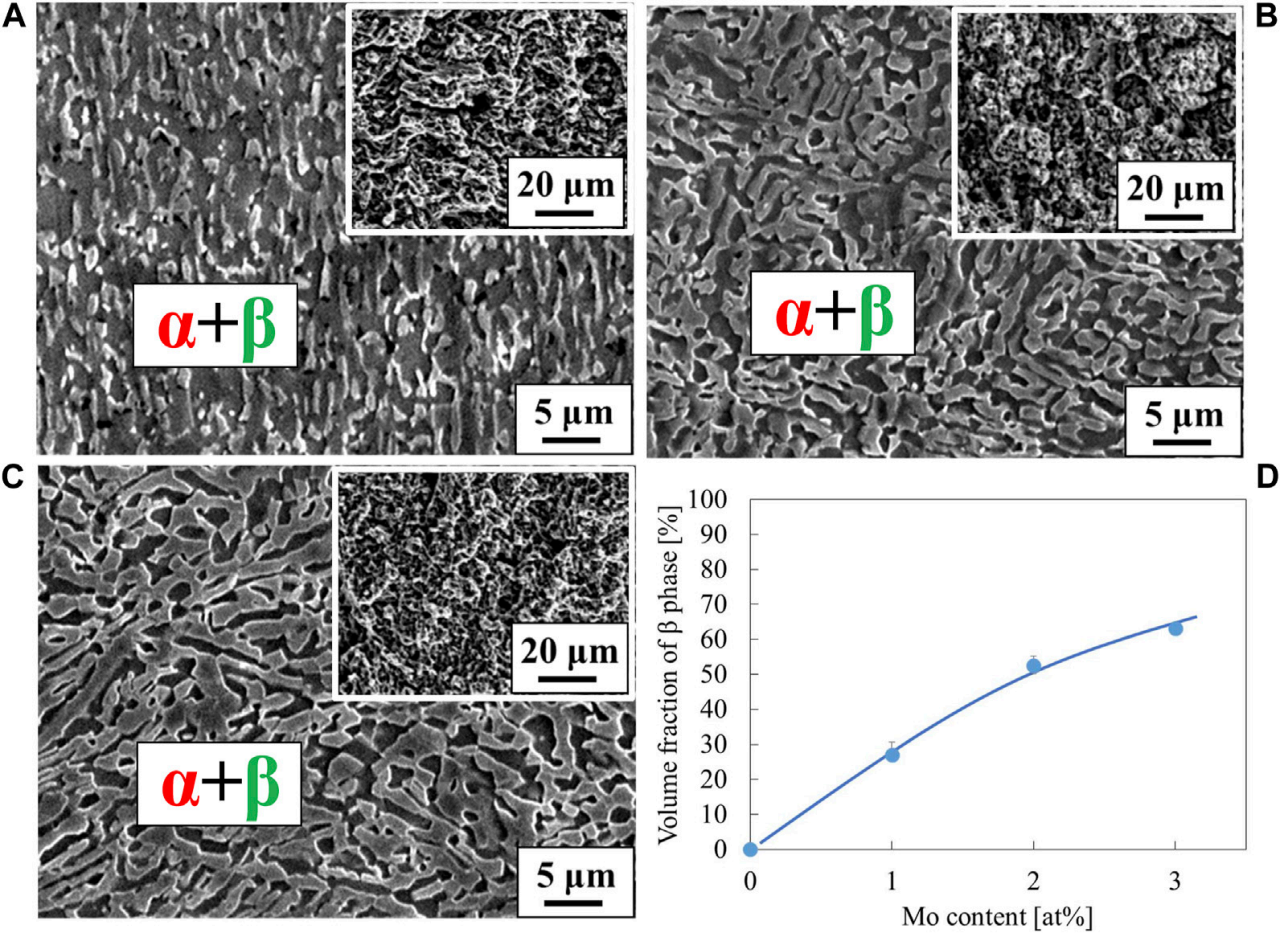
Scanning electron micrographs for the specimens; **(A)** Ti-1.0Mo-3.0O, **(B)** Ti-2.0Mo-3.0O, and **(C)** Ti-3.0Mo-3.0O. The insert image at the upper right corner of each image is a scanning electron micrograph for the fracture surface. The volume fraction of β phase was plotted in the inserted graph **(D)**.

The stress-strain curves of Ti-(1.0, 2.0, 3.0)Mo-3.0O alloys are shown in Figure 9. The tensile strength of Ti-(1.0, 2.0, 3.0)Mo-3.0O alloys were 1,297, 1,325, and 1,313 MPa, where the total strain were 15.5, 9.0, and 4.1%, respectively. Adding 3.0 at% oxygen increased the tensile strengths of Ti-(1.0, 2.0, 3.0)Mo alloys, and the increment values of tensile strength were 640, 423, and 286 MPa, respectively. Although there was almost no change in tensile strength as the content of Mo increased, a decrease in the total strain was observed. The Ti-(1.0, 2.0)Mo-3.0O alloys exhibited uniform and small local deformation, while the Ti-3.0Mo-3.0O alloy only showed uniform deformation before fracture. We should emphasize that Ti-1.0Mo-3.0O alloy exhibited an excellent tensile strength of 1,297 MPa and a sufficient total strain of 15.5%. Figure 10 shows the XRD profiles of Ti-yMo-3.0O alloys; (A) y = 1.0, (B) y = 2.0, (C) y = 3.0. Diffraction peaks of α, α′ phase (hcp), and β phase (bcc) were detected in all samples. Figure 11 shows scanning electron micrographs (backscattered electron image: BEI) for the Ti-(1.0, 2.0, 3.0)Mo-3.0O alloys. In all samples, a fine two-phase structure of several micrometers was confirmed. The graph in Figure 11D shows the variation of the β phase volume fraction in the BEI, indicating that the β phase fraction increased as molybdenum content increased. Furthermore, by comparing Figure 8, oxygen addition suppressed the β phase fraction and increased the α phase fraction. The insert image at the upper right corner of each image is the scanning electron micrograph for the fracture surface of each sample. The fracture surface of each sample exhibited a very fine dimple pattern of several micrometers in diameter, whose diameter was almost the same as that of the grain size of α+β two-phase mixture. Although the dimple size of Ti-(1.0, 2.0, 3.0)Mo-3.0O alloys was almost the same as those of Ti-(1.0, 2.0, 3.0)Mo-1.5O alloys, the dimple for Ti-(1.0, 2.0, 3.0)Mo-3.0O alloys seemed to be more flaky.

## 4 Discussion

### 4.1 Microstructure formation in the hot-rolled Ti-(0, 1.0, 2.0, 3.0)Mo-(0, 1.0, 2.0, 3.0)O alloys

**FIGURE 12:**
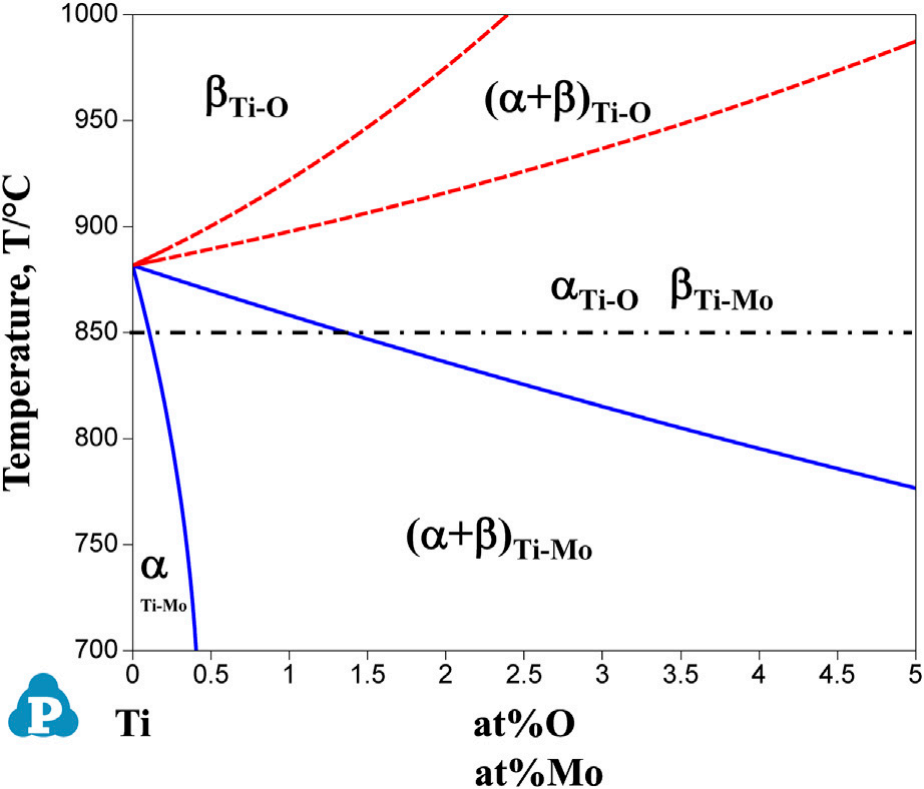
Overlapped graph of calculated phase diagrams of Ti-Mo and Ti-O binary system. Solid and dotted curves represent the phase boundaries of Ti-Mo system and Ti-O system, respectively. The dot-dash line at 850°C indicates the hot-rolling temperature.

Since mechanical properties are highly dependent on the microstructure, we first consider the formation of the microstructure of each sample after the hot-rolling process. Figure 12 shows the phase diagram calculations for Ti-Mo and Ti-O systems superimposed on one drawing. The solid and dash curves for phase boundaries are for the Ti-Mo and Ti-O systems. When molybdenum, a β-phase stabilizing element, is added to the Ti, the phase boundary moves toward the low-temperature side, and the β-phase region becomes wider. On the other hand, when oxygen, which is an α-phase stabilizing element, is added to the Ti, the phase boundary moves toward the high-temperature side, and the α-phase region becomes wider. Based on this phase diagram, we first consider the microstructures formed after the hot-rolling process of binary Ti-O and Ti-Mo alloys.

In the Ti-(0, 1.5, 3.0)O alloys, equiaxed grains of the α phase were observed in all samples, as shown in Figure 2. From the Ti-O binary phase diagram in Figure 12, all samples of any oxygen concentration should have the single α phase at the rolling temperature of 850°C. Therefore, the equiaxed grains in Ti-(0, 1.5, 3.0)O alloys were formed due to the heat treatment in the single α phase region. Furthermore, the grains became finer with the addition of oxygen in the alloys. This is because the oxygen, which tends to segregate at grain boundaries, suppresses the movement of grain boundaries and suppresses grain growth during the hot-rolling process. Syarif et al. (2013) evaluated the segregation amount of oxygen on the grain boundary at 1,000°C for the Ti-10Mo alloy based on the Seah and Hondros’ segregation equation. They calculated the concentration of oxygen segregation on the grain boundaries as 45 at%O. Kawano et al. (2019) also showed that the grain growth of β phase was suppressed by the oxygen addition in Ti-14Nb alloys.

On the other hand, in the Ti-1.0Mo and Ti-2.0Mo alloys, fine grains of several micrometers in size were obtained, as shown in Figures 5A, B. In addition, in the Ti-3.0Mo alloy, equiaxed grains of approximately 5–20 μm in diameter were observed, as shown in Figure 5C. From the Ti-Mo phase diagram in Figure 12, the β transus temperatures of the Ti-(1.0, 2.0, 3.0)Mo alloys are approximately 860°C for the Ti-1.0Mo alloy, and about 840°C and 810°C for the Ti-2.0Mo and Ti-3.0Mo alloys, respectively. In other words, based on this phase diagram, when performing the hot-rolling at 850°C, Ti-1.0Mo alloy would be hot-rolled in the α+β two-phase region, while Ti-2.0Mo and Ti-3.0Mo alloys would be hot-rolled in the single β phase region. However, the microstructure of Ti-1.0Mo and Ti-2.0Mo alloys were very similar, while that of Ti-3.0Mo differed. The possible reason is that all the Ti alloys contain oxygen, as shown in Table 1, and the oxygen should change the phase stability and phase transformation of Ti-Mo alloys (Chou, 2021; Chou and Marquis, 2022). Table 2 shows the calculation results of the mole fraction of the equilibrium phase and the molybdenum and oxygen concentrations of each phase at 850°C for the alloys concerning the actual oxygen content of each alloy. The thermodynamic data file (TDB file) for Ti-Mo-O ternary phase calculations was created by referring to the TDB files for the binary Ti-Mo, Ti-O, and Mo-O systems (Lee and Saunders, 1997; Saunders, 1998; Zhang et al., 2014). Only α and β phases were taken into account for the calculation. Table 2 shows that the Ti-2.0Mo alloy containing 0.26at% oxygen consists of α+β two-phase at 850°C. Furthermore, it also suggests that the Ti-3.0Mo containing 0.31at% oxygen alloy may have the α+β two-phase mixture, but the β phase fraction is 91.4%, which is quite close to the β single-phase. In this calculation, the amount of Mo content in an alloy was supposed to be the nominal composition. If the actual Mo concentration of Ti-3.0Mo could be a little higher than 3.0at%Mo, the nominal composition of Ti-3.0Mo alloy would be in the β single-phase region at 850°C. Therefore, Ti-1.0Mo and Ti-2.0Mo alloys were hot-rolled in the α+β two-phase region, and Ti-3.0Mo alloy was hot-rolled in the β single-phase region. Consequently, Ti-1.0Mo and Ti-2.0Mo alloys exhibited similar fine-grain microstructures, while Ti-3.0Mo exhibited different ones. It should be noted that the XRD results showed the Ti-(1.0, 2.0, 3.0)Mo alloys exhibited a single hcp phase. Kaneko and Huang (Kaneko and Huang, 1963) showed that the Ms points of Ti-Mo binary alloys are approximately 750°C, 680°C, and 600°C for Ti-1.0Mo, Ti-2.0Mo, and Ti-3.0Mo alloys, respectively. Therefore, whether the samples being hot-rolled in the α+β two-phase region or hot-rolled in the β-single phase region, after cooling to room temperature, the β phase could not remain during cooling; instead, the martensitic transformation from β to α′ phase would occur. In the end, the crystal structure of Ti-(1.0, 2.0, 3.0)Mo alloys exhibited α or α′ hcp phase.

**TABLE 2:** Equilibrium phase calculation of samples used in the present study and Ti-(1, 2, 3)Mo imaginary pure binary system without oxygen.

Nominal composition (at%)	Oxygen content (at%)	α phase fraction (%)	at%MoIn α phase	at%OIn α phase	β phase fraction (%)	at%Mo in beta	at%O in beta
Ti	0.56	100.0	0.00	0.56	0.0		
Ti-1.5O	1.61	100.0	0.00	1.61	0.0		
Ti-3.0O	2.96	100.0	0.00	2.96	0.0		
Ti-1.0Mo	0.22	43.1	0.10	0.42	56.9	1.68	0.07
Ti-2.0Mo	0.26	12.7	0.09	1.12	87.3	2.28	0.13
Ti-3.0Mo	0.31	8.6	0.07	2.06	91.4	3.28	0.14
Ti-1.0Mo-1.5O	1.62	72.2	0.06	2.19	27.8	3.43	0.14
Ti-2.0Mo-1.5O	1.57	53.6	0.05	2.83	46.4	4.25	0.12
Ti-3.0Mo-1.5O	1.66	44.6	0.03	3.62	55.4	5.39	0.08
Ti-1.0Mo-3.0O	2.87	81.4	0.04	3.51	18.6	5.21	0.09
Ti-2.0Mo-3.0O	2.95	68.9	0.02	4.26	31.1	6.37	0.06
Ti-3.0Mo-3.0O	3.03	60.4	0.02	4.99	39.6	7.56	0.03
Ti-1.0Mo	0.00	29.2	0.11	0.00	70.8	1.37	0.00
Ti-2.0Mo	0.00	0.0			100.0	2.00	0.00
Ti-3.0Mo	0.00	0.0			100.0	3.00	0.00

**FIGURE 13:**
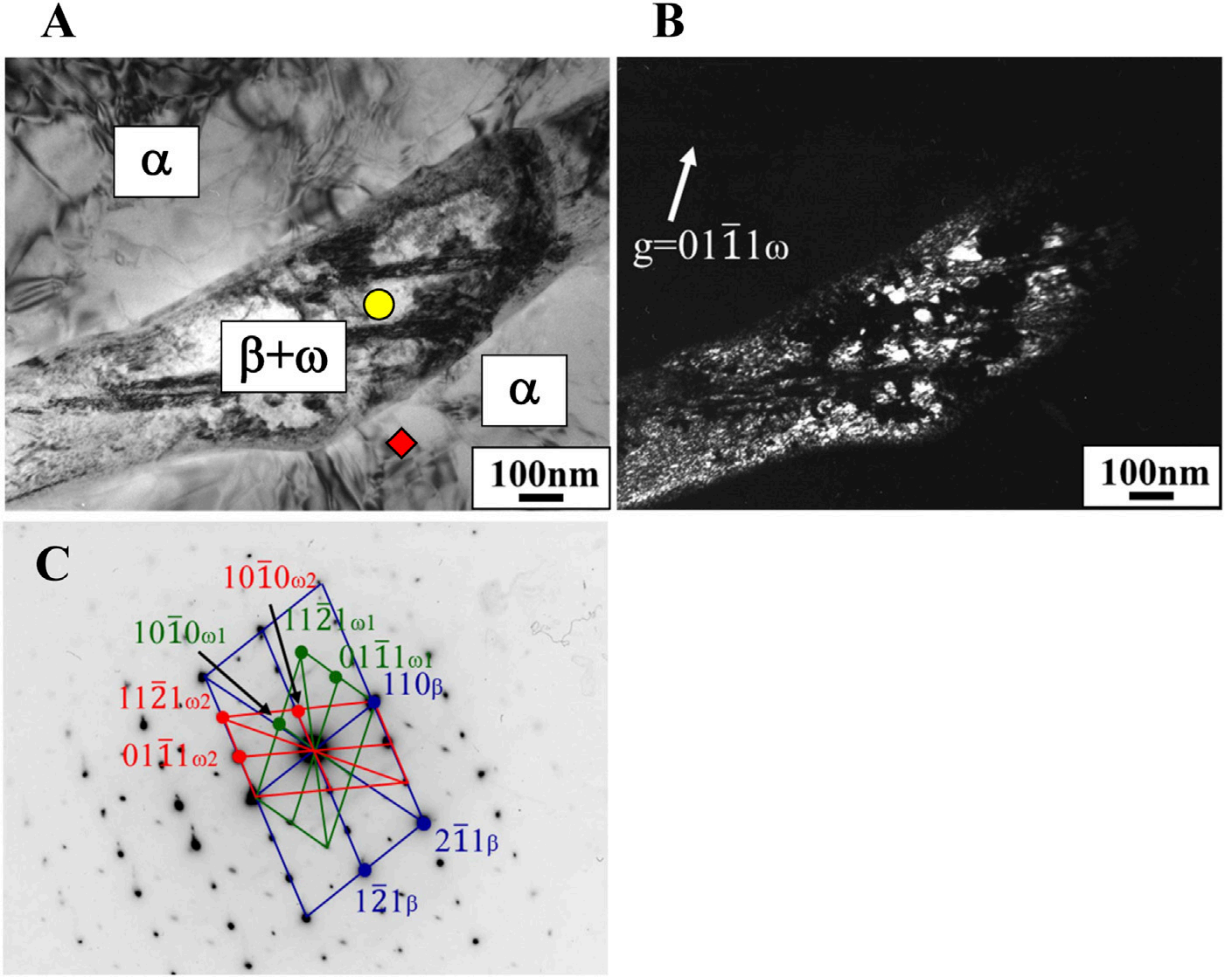
TEM micrographs of Ti-1.0Mo-3.0O alloy; **(A)** bright field image, **(B)** dark field image of omega phase reflection, and **(C)** selected electron diffraction pattern with pattern index.

**FIGURE 14:**
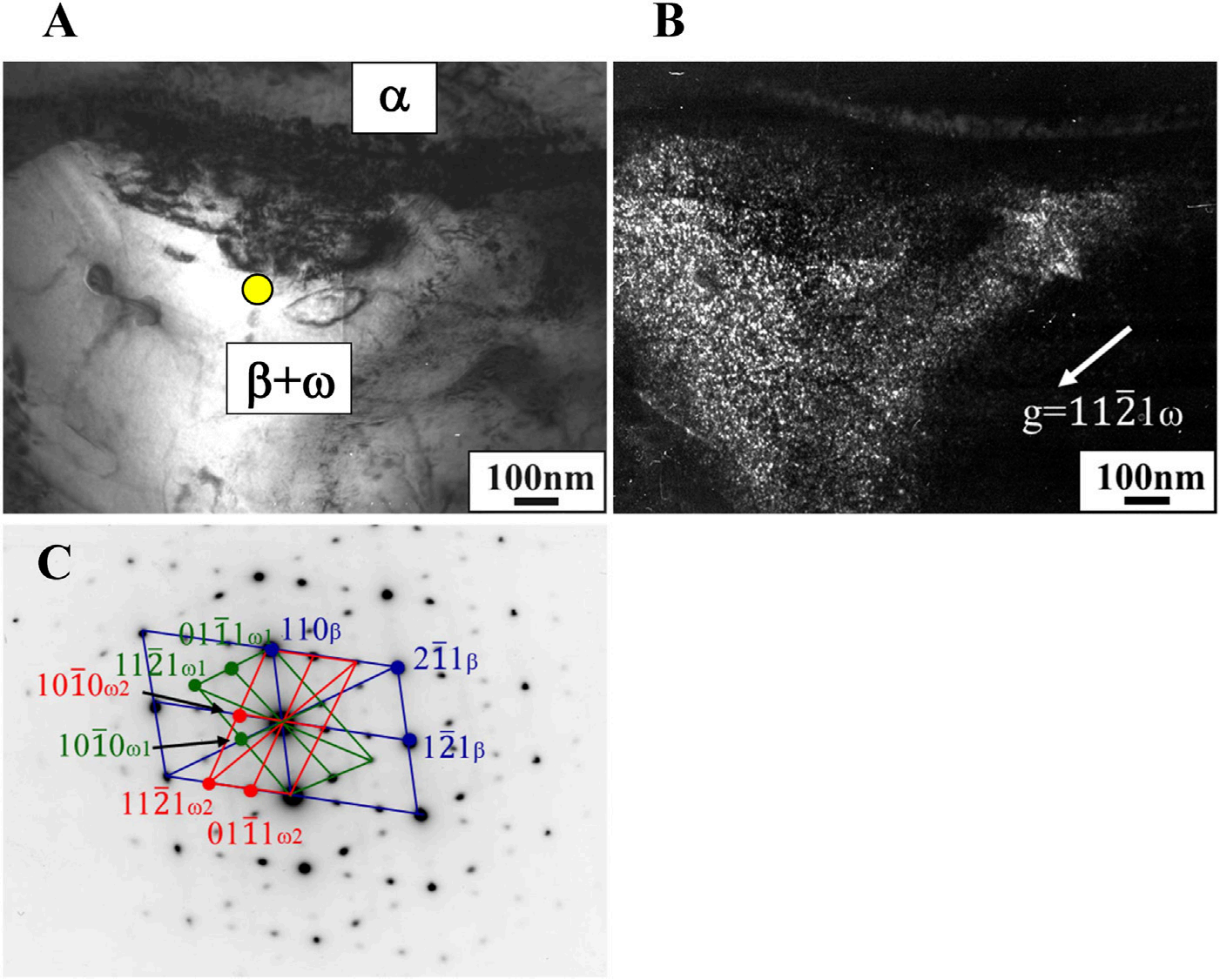
TEM micrographs of Ti-3.0Mo-3.0O alloy; **(A)** bright field image, **(B)** dark field image of omega phase reflection, and **(C)** selected electron diffraction pattern with pattern index.

In the Ti-(1.0, 2.0, 3.0)Mo-(1.5, 3.0)O alloys, an α+β two-phase mixture was confirmed in all samples, as shown in Figures 7, 10. The reason why the β phase remained without undergoing martensitic transformation in the cooling after hot-rolling can be explained regarding the Mo concentration of the β phase of each alloy shown in Table 2. The equilibrium Mo content in the β phase of Ti-(1.0, 2.0, 3.0)Mo-(1.5, 3.0)O alloys are 3.43–7.56 at%. The Mo content in the β phase increases, increasing not only an alloy’s molybdenum content but also the alloy’s oxygen content. The previous studies showed that the β to athermal ω phase transformation (Nejezchlebov et al., 2016) started at about four at%Mo during quenching, forming β+ω (athermal) (Murray, 1981). There is no good agreement about the maximum Mo content at which the athermal ω phase is formed. However, some researchers showed the upper concentration of Mo for β to athermal ω phase formation was around 10at%Mo (Murray, 1981). In fact, the transmission electron microscopy observation revealed that β grains of the Ti-1.0Mo-3.0O alloy contained very fine athermal ω particles, as shown in Figure 13. Figure 13 is TEM micrographs for the Ti-1.0Mo-3.0O alloy; (A) bright field image, (B) dark field image of ω phase showing the fine athermal ω particles, (C) selected area diffraction pattern revealing the coexistence of β and ω phases. The particle size of the ω phase was around 10 nm in diameter. The Mo content in α and β phases in the Ti-1.0Mo-3.0O alloy was evaluated using EDS equipped with TEM, where the measured positions were indicated by the circle and diamond marks in Figure 13A. The values of Mo content in α and β phases in the Ti-1.0Mo-3.0O alloy were 0.13 and 5.58 at%, respectively, close to those of calculated values in Table 2. Figure 14 is TEM micrographs for the Ti-3.0Mo-3.0O alloy; (A) bright field image, (B) dark field image of ω phase showing the very fine athermal ω particles, (C) selected area diffraction pattern revealing the coexistence of β and ω phases. The particle size of the ω phase was around 5 nm in diameter. The Mo content in β phase in the Ti-3.0Mo-3.0O alloy was evaluated using EDS equipped with TEM, where the measured position was indicated by the circle mark in Figure 14A. The values of Mo content in β phase in the Ti-3.0Mo-3.0O alloy was 7.41 at%, close to the calculated values in Table 2. The size of athermal ω particles in β phase in the Ti-3.0Mo-3.0O alloy was much smaller than that in the Ti-1.0Mo-3.0O alloy due to the increase of Mo content in β phase. As the Mo concentration in the β phase increased, the β phase became more stable, and the transformation temperature of the ω phase decreased, resulting in the formation of finer ω phase particles.

It can be concluded that by adding oxygen to the Ti-Mo system, the α+β two-phase region expanded, resulting in the formation of fine α+β grains in the hot-rolled Ti-(1.0, 2.0, 3.0)Mo-(1.5, 3.0)O alloys at 850°C.

### 4.2 Mechanical properties of the hot-rolled Ti-(0, 1.0, 2.0, 3.0)Mo-(0, 1.0, 2.0, 3.0)O alloys

Table 3 summarizes the mechanical properties of the alloys used in the present study. We checked the reproducibility of the tensile test for three samples of the Ti-1Mo-3.0O alloy. Variations of 0.2% proof strength and tensile strength were around several percent. On the other hand, fracture and total strains exhibited relatively large variations of 28% and 23%, respectively.

**TABLE 3:** Mechanical properties of samples used in the present study. (NA: not applicable).

Nominal composition (at%)	Oxygen content (at%)	Vickers hardness [Hv]	0.2% proof strength (σ_0.2_)	Tensile strength (σ_max_)	Fracture strain [%]	Total strain [%]
Ti	0.56	171	331	464	29.0	29.2
Ti-1.5O	1.61	308	722	828	13.8	14.5
Ti-3.0O	2.96	400	NA	NA	0.0	0.0
Ti-1.0Mo	0.22	219	463	657	23.4	24.0
Ti-2.0Mo	0.26	290	801	902	11.3	12.0
Ti-3.0Mo	0.31	350	926	1,027	8.0	9.1
Ti-1.0Mo-1.5O	1.62	342	947	1,048	22.8	23.5
Ti-2.0Mo-1.5O	1.57	339	1,004	1,153	24.7	25.6
Ti-3.0Mo-1.5O	1.66	364	974	1,122	19.4	20.2
Ti-1.0Mo-3.0O	2.87	411	1,271	1,297	14.4	15.5
Ti-2.0Mo-3.0O	2.95	407	1,299	1,325	7.9	9.0
Ti-3.0Mo-3.0O	3.03	410	1,246	1,313	3.1	4.1

In the Ti-(0, 1.5, 3.0)O alloys, which had α-phase equiaxed grains, an increase in Vickers hardness and tensile strength and a decrease in the total strain were confirmed with an increase in the oxygen content of the alloys. In addition, no plastic deformation was observed in the Ti-3.0O alloy, exhibiting a brittle fracture surface. There are three types of slip systems in the α-phase: the prismatic slip $\left\{1\bar{1}00\right\}\langle 11\bar{2}0\rangle$ , the conical slip $\left\{1\bar{1}01\right\}\langle 11\bar{2}0\rangle$ , and the basal slip $\left\{0001\right\}\langle 11\bar{2}0\rangle$ . In these slip systems, the slip direction is limited in the basal plane, and no slip occurs in the c-axis direction. Therefore, in order for a polycrystalline titanium alloy with α phase to undergo plastic deformation while maintaining compatibility between each grain, deformation twinning or <c+a> slip in which the Burgers vector has a component in the c-axis direction must occur (Churchman, 1954; Akhtar, 1975; Yoo, 1981). Murayama et al. reported that the emerging frequency of deformation twins decreased as oxygen content increased, and almost no deformation twins were observed in the Ti-0.4 mass%O (Ti-1.2at%O) alloy (Murayama et al., 1993). In high oxygen-containing alloys, <c+a> slip is activated instead of deformation twins for the deformation in the c-axis direction. The < c+a> slip has a high critical resolved shear stress (CRSS), and under conditions where <c+a> slip is active, cleavage failure is likely to occur. In other words, in high oxygen concentration alloys, in addition to solid solution hardening, the elongation is thought to be extremely small due to the activity of <c+a> slip, which tends to cause cleavage fracture. The Ti-3.0O alloy has a higher oxygen content than Ti-0.4 mass%O, which has been reported to have no deformation twins. Therefore, the Ti-3.0O alloy was considered brittle and prone to cleavage fracture.

In the Ti-(1.0, 2.0, 3.0)Mo alloy, the Vickers hardness and tensile strength increased almost proportionally to the Mo content due to the effect of solid solution strengthening, and the total elongation decreased accordingly. There was no significant difference in the fracture surfaces of these Ti-Mo alloys (Figures 5D–F), and it is thought that the increase in solid solution strengthening depending on the Mo content caused these changes in mechanical properties.

The Ti-(1.0, 2.0, 3.0)Mo-(1.5, 3.0)O alloys exhibited higher tensile strength and more significant total strain compared to the Ti-(1.5, 3.0)O alloys at the same oxygen content. Ti-(1.5, 3.0)O alloys were partially or entirely covered with a brittle cleavage surface, but Ti-(1.0, 2.0, 3.0)Mo-(1.5, 3.0)O alloys had fine ductile dimple patterns on the entire fracture surface. Ti-(1.0, 2.0, 3.0)Mo-(1.5, 3.0)O alloys had a fine α+β two-phase structure in all samples, and the formation of such the microstructure prevented the occurrence of cleavage fracture due to oxygen addition. The number of slip systems in the β phase is larger than that in the α phase, so the β phase possesses better plastic workability. Furthermore, β-type Ti alloys with low alloy compositions exhibit significant ductility due to $\left\{332\right\}\langle 11\bar{3}\rangle$ twinning (Hanada and Izumi, 1987; Lai et al., 2016).

A detailed analysis of the plastic deformation behavior of Ti-(1.0, 2.0, 3.0)Mo-(1.5, 3.0)O alloys revealed that the plastic deformation mode differed depending on the alloy composition. In the Ti-(1.0, 2.0, 3.0)Mo-1.5O alloys, deformation due to good uniform elongation accompanied by work hardening was confirmed. Furthermore, in the Ti-1.0Mo-3.0O alloy, deformation progressed under a certain stress after yielding, leading to fracture. On the other hand, although the yield stress of Ti-(2.0, 3.0)Mo-3.0O alloys was not different from that of Ti-1.0Mo-3.0O alloy, their stress decreased immediately after yielding, accompanying local deformation leading to fracture. These changes in the plastic deformation mode may depend on the phase fraction of α and β phases and the Mo concentration in the β phase, which is responsible for ductility. Hida et al. reported that the mechanical properties of β-type Ti-Mo alloys changed significantly depending on the Mo concentration (Hida et al., 1980). They found, in the Ti-14wt%Mo (Ti-7.51at%Mo) alloy, that $\left\{332\right\}\langle 11\bar{3}\rangle$ twins were formed during deformation and exhibited good ductility with work hardening. On the other hand, in the Ti-20wt%Mo (Ti-11.09at%Mo) alloy, which has a thermally more stable β phase than that in the Ti-14wt%Mo alloy, the deformation is localized due to $\left\{112\right\}\langle 11\bar{1}\rangle$ slip after yielding, resulting in no work hardening and low ductility. Comparing the deformation behavior of these Ti-Mo binary alloys with the Ti-Mo-O ternary alloy, we find that the deformation behavior of the Ti-(1.0, 2.0, 3.0)Mo-1.5O alloy is similar to that of the Ti-14wt%Mo alloy. We also see the deformation behavior of the Ti-(2.0, 3.0)Mo-3.0O alloy is likely to be that of the Ti-20wt%Mo alloy. The case of the Ti-1.0Mo-3.0O alloy is considered to be in an intermediate situation between them. The Ti-(1.0, 2.0, 3.0)Mo-1.5O and Ti-1.0Mo-3.0O alloys that showed good ductility may have low thermal stability β phase with excellent deformability. As shown in Table 2, the Mo concentration in β phase of the α+β two-phase mixture increases as the content of Mo and oxygen in the alloy increases. Therefore, in oxygen-added Ti alloys, it is essential to control not only the formation of an α+β microstructure and the appropriate volume fractions of α and β phases but also the Mo concentration in the β phase of the α+β two-phase mixture.

In conclusion, the formation of the α+β two-phase mixture, in which the α phase solid-solution strengthened by oxygen and the β phase with excellent deformability coexist, is highly essential for the development of the high strength, high hardness, and high ductility of Ti alloys containing high amount of oxygen.

## 5 Conclusion

We examined the effects of oxygen addition on the microstructure formation and mechanical properties of the hot-rolled Ti-Mo alloys and obtained the following results.

(1) The constituent phase of Ti-(1.5, 3.0)O alloys and Ti-(1.0, 2.0, 3.0)Mo alloys was α or α′ phase with hcp structure. As the amount of alloying elements in these alloys increased, Vickers hardness and tensile strength increased, and the total strain decreased. In the Ti-3.0O alloy, plastic deformation could not be confirmed due to cleavage fracture at the initial deformation stage.
(2) All Ti-(1.0, 2.0, 3.0)Mo-(1.5, 3.0)O ternary alloys had α+β two phase mixture. By comparing the fact that the constituent phase of the Ti-(1.0, 2.0, 3.0)Mo alloys was α+α′ or α′ phase, the oxygen contributed to the formation of the β phase in Ti-Mo-O ternary alloys. From calculating the equilibrium composition of phases in Ti-Mo-O alloys, we find that the oxygen addition increases the Mo concentration in the β phase of the α+β two-phase mixture at hot-rolling temperature, at 850°C. The increase in Mo concentration in the β phase due to oxygen addition stabilized the β phase against the martensitic transformation of the β phase during cooling after hot rolling.
(3) The developed Ti-Mo-O ternary alloys with α+β phases exhibited a good balance of tensile strength and total strain, e.g., the tensile strength and total strain of Ti-2.0Mo-1.5O alloy were 1,153 MPa and 25.6%. The strength and hardness of Ti-Mo-O ternary alloys are determined not only by the phase fraction of α and β phases but also by the strengthening and hardening of both the α phase by solute oxygen and β phase by solute molybdenum. In addition, the ductility of Ti-Mo-O alloys would be changed depending on the concentration of Mo in the β phase that contributes to elongation. The β phase, with a relatively low Mo concentration and low thermal stability, has better ductility. In other words, in order to develop a low-cost, high-hardness, high-strength, and high-ductility alloy using oxygen, it is necessary to form a fine α+β two-phase mixture and appropriately adjust the ratio of α and β phases. Furthermore, controlling the oxygen and Mo concentrations in the α and β phases is essential.
(4) By comparing the tensile strength and total strain of a Ti-6Al-4V ELI, a typical biomedical Ti alloy, i.e., about 860 MPa and 15%, the developed Ti-(1.0, 2.0, 3.0)Mo-1.5O alloys and Ti-1.0Mo-3.0O alloy are superior in mechanical properties to Ti-6Al-4V ELI alloy. Therefore, they would be candidates for a biomedical Ti alloy.

## Data Availability

The original contributions presented in the study are included in the article/Supplementary material, further inquiries can be directed to the corresponding author.
